# Right lateral geniculate nucleus infarct presenting as a left monocular temporal hemianopia

**DOI:** 10.1002/ccr3.2189

**Published:** 2019-05-14

**Authors:** Antonio Liu, Luis Sanguino

**Affiliations:** ^1^ Department of Neurology California Hospital Medical Center Los Angeles California; ^2^ Graduate Medical Education California Hospital Medical Center Los Angeles California

**Keywords:** bilateral congruous homonymous hemianopia, lateral geniculate nucleus, left temporal monocular hemianopia, visual field defects, visual field pathway

## Abstract

A lesion to the right lateral geniculate nucleus should cause a complete left monocular hemianopia. We present a case of a patient with left eye monocular temporal hemianopia with two MRIs demonstrating an ischemic defect at the right lateral geniculate body as the likely culprit. Patient improved after 4 days.

## 1 INTRODUCTION

An elderly male patient presents with left eye monocular temporal hemianopia. Careful repeated examinations by primary care physician, neurologist, and ophthalmologist confirmed the monocular nature of the finding. A small ischemic lesion at or near the right lateral geniculate body on two separate MRIs is identified as the sole possible explanation. Symptoms resolved after four days.

Monocular hemianopia is defined as the loss of the temporal or nasal visual field in one eye, which usually involves a specific lesion anterior to the optic chiasm (prechiasmal). Additionally, a lesion at the right lateral geniculate nucleus (postchiasmal) is expected to cause a binocular homonymous left hemianopia. Any lesion posterior to the optic chiasm is expected to affect both eyes at the same time. Our patient has presented with a left eye monocular temporal hemianopia, which was confirmed by multiple clinicians’ careful detailed examination sometimes repeatedly in the same day. Patient was always fully compliant, and his findings were almost always consistent. Although a few ischemic lesions are identified on two separate MRIs three days apart, three radiologists independently pointed out that there was a subtle small ischemic lesion at or around the right LGN. Since no other lesion will produce a visual deficit/findings, the relationship between his vision complaint and this subtle right LGN lesion must be correlated and, hence, investigated. The LGN is comprised of six different layers, of which three (layers 2, 3, and 5) receive information from the ipsilateral eye and the other three (layers 1, 4, and 6) receive information from the contralateral eye. In this report, a case of a left monocular temporal hemianopia thought to be due to a partial infarct to the right lateral geniculate nucleus or a small infarct affecting the pathways leading to layers 1, 4, and 6 will be presented. The anatomy and vascular supply of the LGN as well as the different visual defects caused by lesions to the LGN will also be reviewed.

## 2 CASE REPORT

The patient is a 61‐year‐old Korean male with a significant past medical history of rheumatoid arthritis, hypertension, and diabetes mellitus who presented to the hospital with sudden onset of blurry vision in his left eye three days prior. A head CT had been performed at an outside facility, which was negative for acute ischemia, hemorrhage, midline shift, or extra‐axial fluid collection. The patient denied visual complaints in his right eye and had no history of similar events.

Ophthalmologic and Neurologic consultations initially revealed visual acuity was 20/40 on right eye; however, on the left eye, there was an apparent left‐sided visual loss. Patient's intraocular pressure was normal in both eyes, pupils were equally round and reactive to light, and no afferent pupillary defect was observed. Moreover, the extraocular muscles were intact and with full range of motion. In the right eye, visual fields were full to finger count. In the left eye, the acuity in the nasal visual field was greater than that in the temporal visual field. In the nasal visual field, the patient was able to count fingers; however, in the temporal visual field, the patient could barely detect gross hand movement at one foot out. External examination was within normal limits, and pen light examination was only remarkable for nuclear sclerotic cataracts bilaterally. Dilated fundus examination demonstrated no evidence of pathology to the vitreous, optic nerve, or retina that might explain the vision loss. Four days later, repeat examination by Ophthalmology demonstrated a stable right eye; nonetheless, the left eye temporal visual field was 20/25 and patient was able to count fingers without mistake on left visual field. Pupil and retina examination were unchanged from previous examination. However, visual field mapping could not be done as this patient was seen in the inpatient setting.

**Figure 1:**
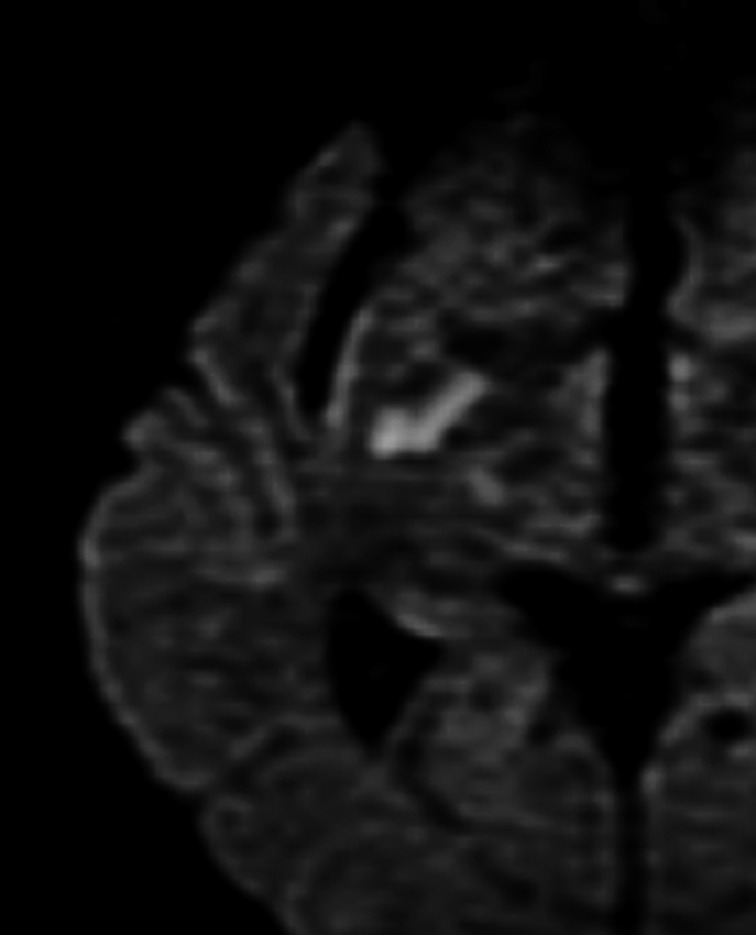
Small ischemic strokes seen on DWI MRI affecting inferior posterior basal ganglia as well as a smaller right lateral inferior posterior thalamus at the vicinity of LGN

Consequently, an initial head MRI with and without contrast was performed, which showed focal areas of restricted diffusion in the right medial temporal lobe, inferior right basal ganglia with possible involvement of the right lateral geniculate nucleus. The head MRI did not show any other pathology such as masses or hemorrhages (Figure 1). The subsequent head MR angiogram done demonstrated no focal occlusion or stenosis, and the MRI of the orbits showed no focal defects bilaterally. At this point, it was evident that the patient was suffering from a left temporal monocular hemianopia and that there must be a correlation with the subtle but definitely present right LGN ischemic lesion. An infarct affecting the right lateral geniculate nucleus would most likely cause bilateral left homonymous hemianopia. Nevertheless, a lesion at the vicinity of right LGN that had taken out the inputs going to layers 1, 4, and 6 became generally accepted among all physicians involved at this point in the care of this patient as the only possible explanation. Patient's left visual symptoms slowly started to improve, and after four days of hospitalization and monitoring, patient's left monocular temporal hemianopia had almost resolved and his vision had almost returned to his normal baseline.

## 3 CASE DISCUSSION

In order to thoroughly comprehend the cause of our patient's left temporal monocular hemianopia, it is extremely important to understand the complete visual pathway as well as the complex organization and anatomy of the lateral geniculate nucleus (LGN). It is believed that the signals arising from the retinal ganglion cells (RGCs) provide a compact representation of the world as we know it, whereas the primary visual cortex extracts this information and rearranges it to provide a coherent view of what the person is actually seeing. The visual pathway is divided into three different areas: prechiasmal, optic chiasm, and retrochiasmal. The prechiasmal visual pathway is formed by axons extending from the retina and forming the optic nerves. The optic nerves will then join and form the optic chiasm. The retrochiasmal visual pathway is composed of the region between the optic chiasm and the primary visual cortex. Fibers arising from the optic chiasm and extending through the optic tracts, which are supplied mainly by the anterior choroidal artery, travel to the lateral geniculate nucleus (LGN).^1^ The lateral geniculate nucleus (LGN) is a sensory relay structure located in the thalamus. It is the main connection between the retina and optic nerves to the primary visual cortex, which is why it is of extreme importance.^1^

**Figure 2:**
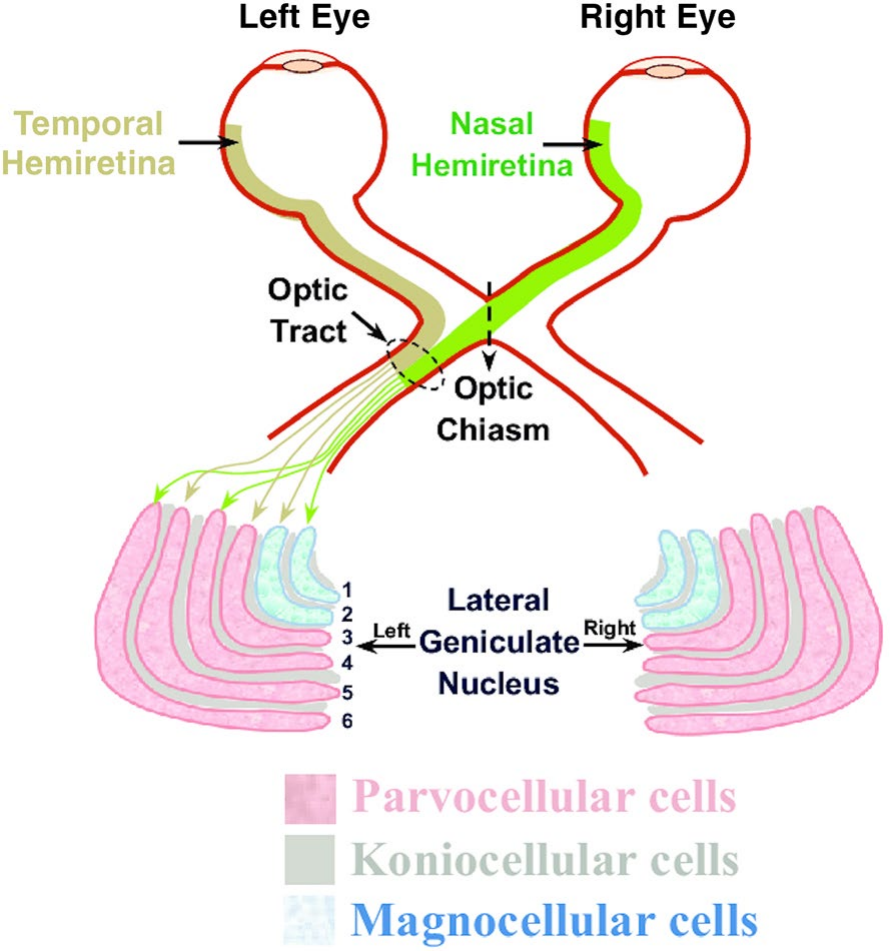
Outline of layers and of organization of P, K, and M cells in the LGN (from Ghodrati et al 2017)^4^

Furthermore, three retinal ganglion cell types (midget, parasol, and bistratified ganglion cells) are very well characterized and have been linked to parallel visual pathways between the retina and primary visual cortex, which remain segregated through the LGN.^2^ The LGN is composed of layers of magnocellular (M cells) and parvocellular cells (P cells), which are enclosed by koniocellular cells (K cells). The inner LGN layers 1 and 2 are comprised of M cells, whereas the outer LGN layers 3, 4, 5, and 6 are comprised of P cells (Figure 2). The K cells are found ventral to the M and P cells. Parasol ganglion cells, which comprise approximately 10% of the cells projecting into the LGN, are considered to give rise to the M cells. Midget cells, on the other hand, are believed to be the origin of P cells and comprise approximately 70% of the total cells projecting to the LGN.^2^ M cells have large receptive fields, fast axonal conduction velocities, and are important for the perception of movement, depth, and changes in brightness. On the contrary, P cells have smaller receptive fields, slower axonal conduction velocities, and are important for the perception of color and form.

**Figure 3:**
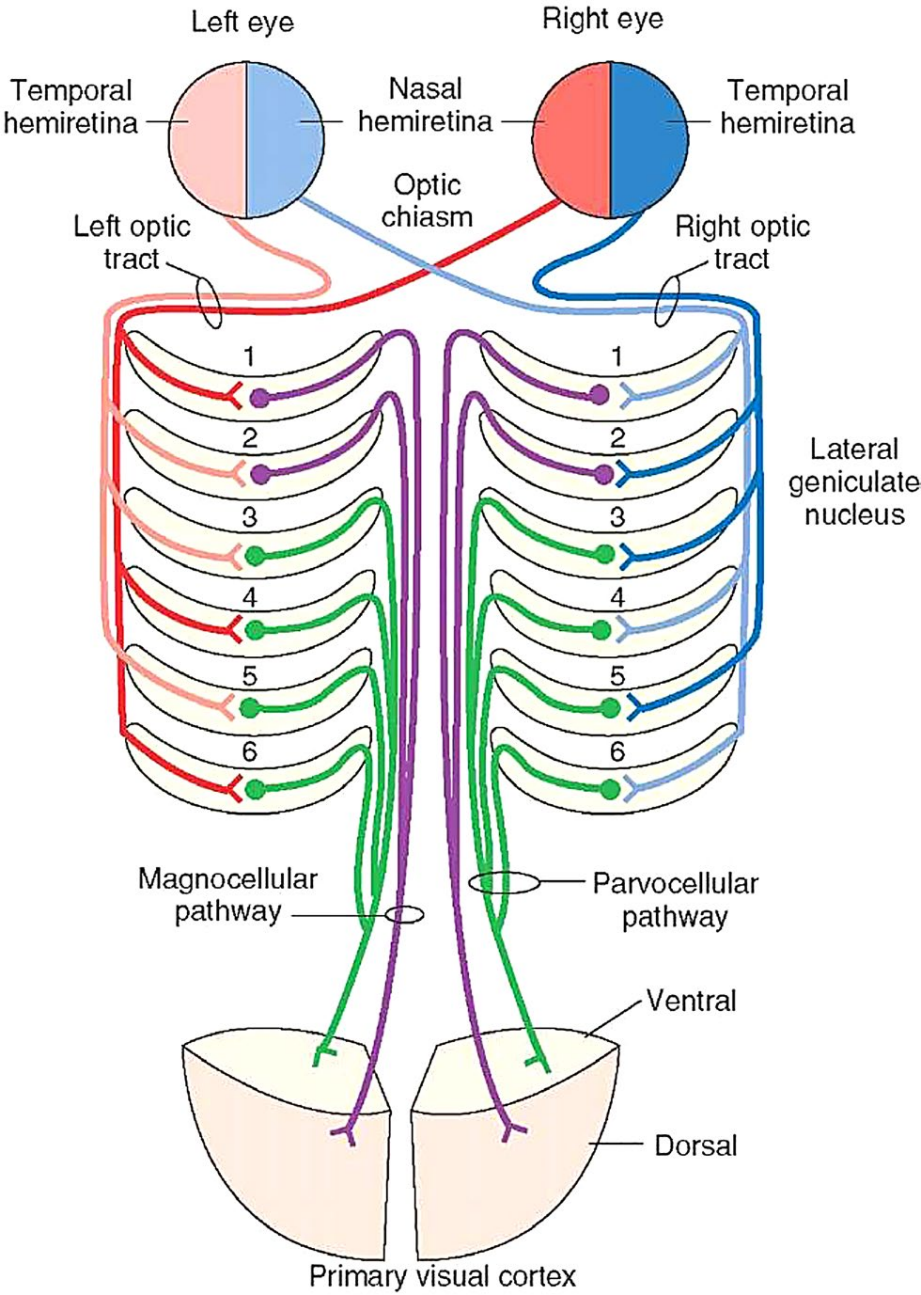
Layers of the lateral geniculate nucleus in processing ipsilateral and contralateral vision (http://www.raynersmale.com/blog/2017/1/26/visual-perception-the-brain)

**Figure 4:**
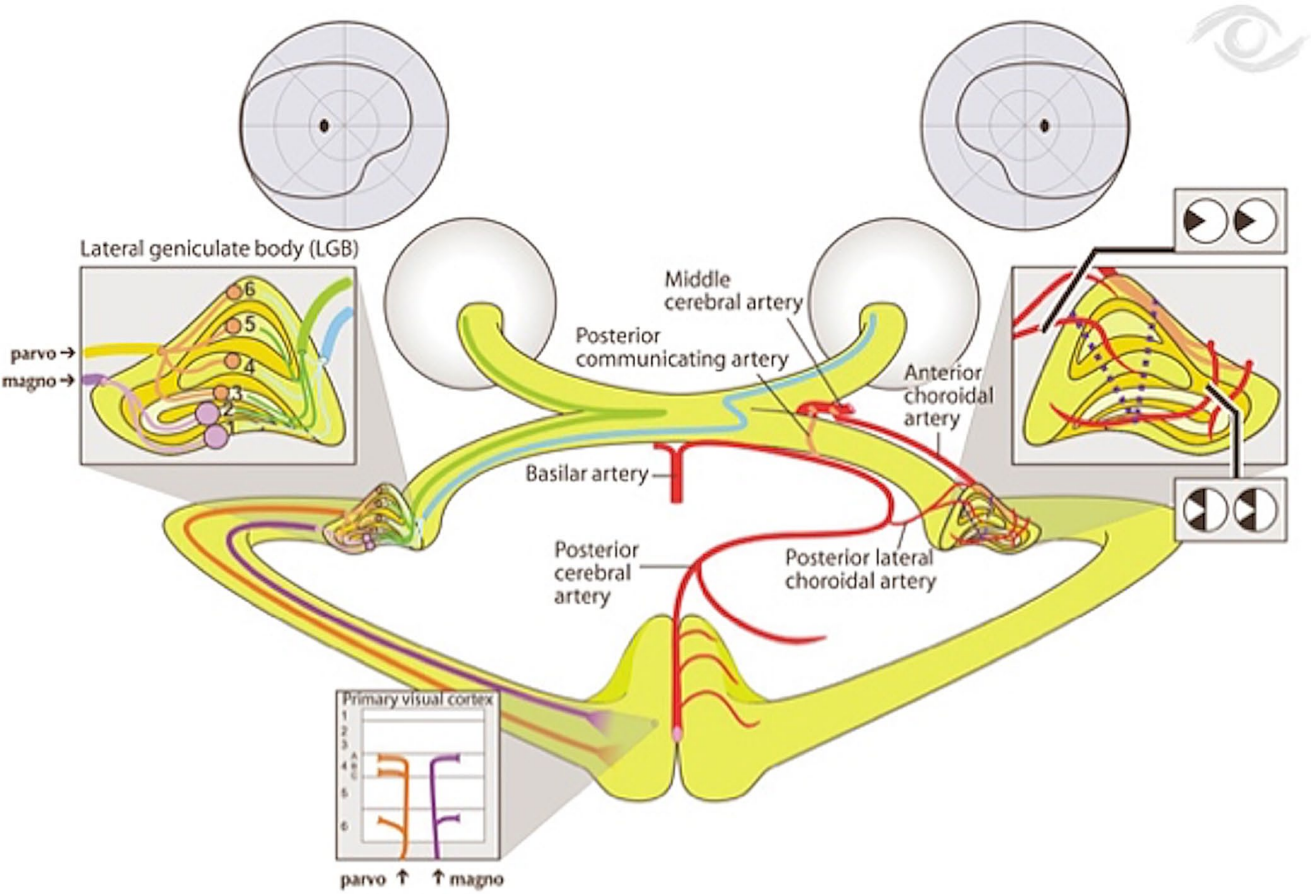
Vascular supply to the layers of the LGN by the anterior choroidal artery and posterior lateral choroidal artery (https://www.clicktocurecancer.info/optic-nerve/iq.html)

Additionally, it is important to note that the eye ipsilateral to the LGN sends information regarding the temporal hemiretina (nasal visual field) to layers 2, 3, and 5, whereas the eye contralateral to the LGN sends information regarding the nasal hemiretina (temporal visual field) to layers 1, 4, and 6 (Figure 3). The patient described above showed a left monocular temporal hemianopia possibly due to a focal partial infarct of the right LGN as per the head MRI (Figure 1). It is likely that this focal infarct in the right LGN affected layers 1, 4, and 6, which would explain the patient's left monocular temporal hemianopia. The LGN has double blood supply from the anterior choroidal artery (a branch from the internal carotid artery) and from the posterior lateral choroidal artery (a branch from the posterior cerebral artery) (Figure 4).^3^ A complete blockage to one of these arteries supplying the LGN would cause a congruous bilateral homonymous hemianopia. A small embolus or thrombus in a smaller branches of these arteries may instead cause smaller and partial visual defects, as seen in our patient.

Luco et al^3^ reports that a complete vascular lesion to the LGN would cause a congruent (same visual field defect in both eyes) wedge‐shaped homonymous hemianopia or a congruent quadrantic contraction of the upper or lower visual field depending on which artery that supplies that LGN is blocked. However, lesions to the LGN are very rare and thus are seldom mentioned in research articles.

Based on the above explanation and discussion, the clinical presentation of this patient, the neuro‐ophthalmologic physical examination, and the head MRI showing a subtle but definite focal ischemic lesion in the vicinity of the right LGN with no other possible explanation of the visual defect seen in this patient, we can conclude that this patient most likely suffered a small stroke at the vicinity of right LGN likely decreasing signaling to layers 1,4, and 6 of the right LGN, which would cause a left temporal monocular hemianopia. Due to the small size of the lesion, authors are not surprised that patient recovered his vision over 4 days.

## CONFLICT OF INTEREST

The authors declare that there is no conflict of interest regarding the publication of this paper.

## AUTHOR CONTRIBUTION

AL and LS: conceived of the presented idea. AL: developed the manuscript's main theory and the main conceptual idea. LS: contributed to the main theory and also worked on the technical details of the manuscript. AL and LS: wrote the manuscript and contributed to the final version of the project.
